# Supplementary material for: An early mechanical coupling of planktonic bacteria in dilute suspensions
Source: Nat Commun. 2017 Aug 9;8:213. doi: 10.1038/s41467-017-00295-z (PMC5548916; doi:10.1038/s41467-017-00295-z)
Supplement: Supplementary file 1 — Supplementary Information [file 41467_2017_295_MOESM1_ESM.pdf]

File name: Supplementary Information
Description: Supplementary Figures and Supplementary Table.

File name: Supplementary Movie 1
Description: Viscoelastic properties of dilute *B. subtilis* wt suspension grown to $OD_{650}$ = 0.5. The initial position of an optically trapped bacterium is shown. When the selected bacterium was moved, the neighboring bacteria in the cluster followed the motion. A long-range coupling of coordinated motion could be observed. The neighboring bacteria in the cluster responded differently to high or low frequency oscillations. Optically trapped bacterium was moved to different spots of the view field which produced similar coupling effects. The extracellular matrix material responsible for the observed viscoelastic effect is not visible. In the absence of motion of optically trapped bacterium, neighboring bacteria in the cluster exhibited the uncorrelated Brownian motion.

File name: Supplementary Movie 2
Description: Viscoelastic properties of dilute *E. coli* bacterial suspension grown to $OD_{650}$ = 0.5. The initial position of the optical trap is shown. The optically trapped bacterium was manually moved. A long-range coupling of bacterial motion in the cluster was observed.

File name: Supplementary Movie 3
Description: Viscoelastic properties of dilute *V. ruber* bacterial suspension grown to $OD_{650}$ = 0.5. The initial position of the optical trap is shown. The optically trapped bacterium was manually moved. A long-range viscoelastic coupling of bacteria, of approximately the same magnitude, was observed in different parts of the view field.

File name: Supplementary Movie 4
Description: Viscoelastic properties of dilute *S. aureus* bacterial suspension grown to $OD_{650}$ = 0.5. The initial position of the optical trap is shown. The optically trapped bacterium was manually moved in different directions. A shorter coupling than in other bacterial cultures was observed.

File name: Supplementary Movie 5
Description: Viscoelastic properties of dilute *P. fluorescens* bacterial suspension grown to $OD_{650}$ = 0.5. The position of the optical trap motion is shown. The optically trapped bacterium was manually moved in different directions at different speeds. A long-range coupling of coordinated motion in bacterial cluster was observed.

File name: Supplementary Movie 6
Description: Viscoelastic properties of dilute *P. stutzeri* bacterial suspension grown to $OD_{650}$ = 0.5. The position of the optical trap motion is shown. The optically trapped bacterium was manually moved. A long-range coupling of motion in bacterial cluster was observed.

File name: Supplementary Movie 7
Description: Viscoelastic properties of dilute *P. aeruginosa* bacterial suspension grown to $OD_{650}$ = 0.2. Lower cell density was used to avoid visible aggregates formation in the mid of the exponential phase. The initial position of the optical trap is shown. Although cell density was lower than in other bacterial suspensions, a rather long-range coupling of coordinated motion was observed.

File name: Supplementary Movie 8
Description: Mechanical coupling of optically trapped *B. subtilis* wt bacterial pairs grown in SYM medium. The actively trapped bacterium was periodically oscillated, whereas the passive bacterium was trapped, positioned, and released after 4 s. A weak mechanical coupling of passive bacterium can be observed.

File name: Peer Review File
Description

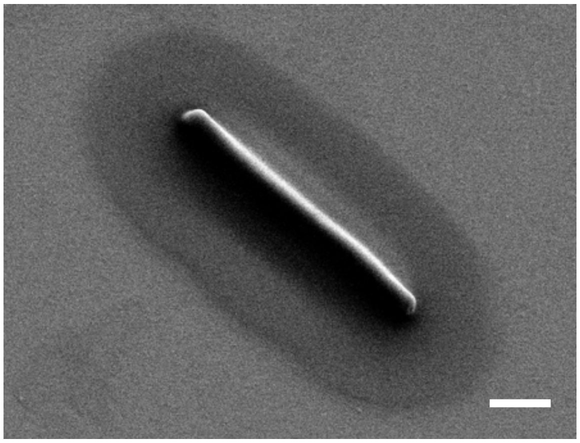

**Supplementary Figure 1. The extracellular material surrounding the *B. subtilis* cell.** SEM micrograph of *B. subtilis* 3610 wt cell in SYM growth medium in the mid of the exponential phase embedded in the extracellular material. Scale bar represents 1 µm.

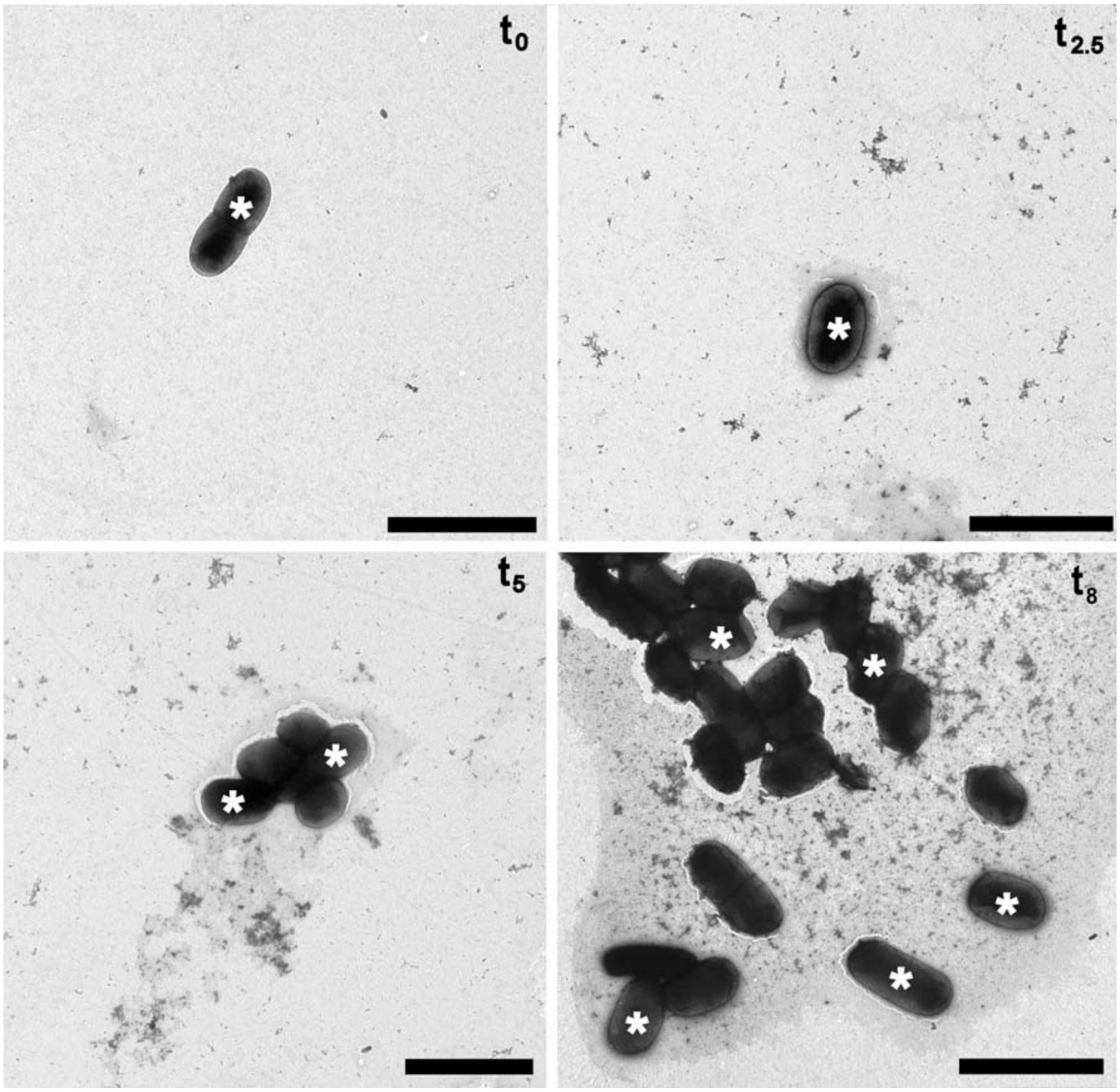


**Supplementary Figure 2. The development of the *E. coli* extracellular network.** TEM micrographs of inoculated growth medium with *E. coli* ($t_0$) and after 2.5, 5, and 8 h of incubation in M9 growth medium. The extracellular material was partially attached to cells (*) or was excreted in the extracellular space. Scale bars represent 3 µm.

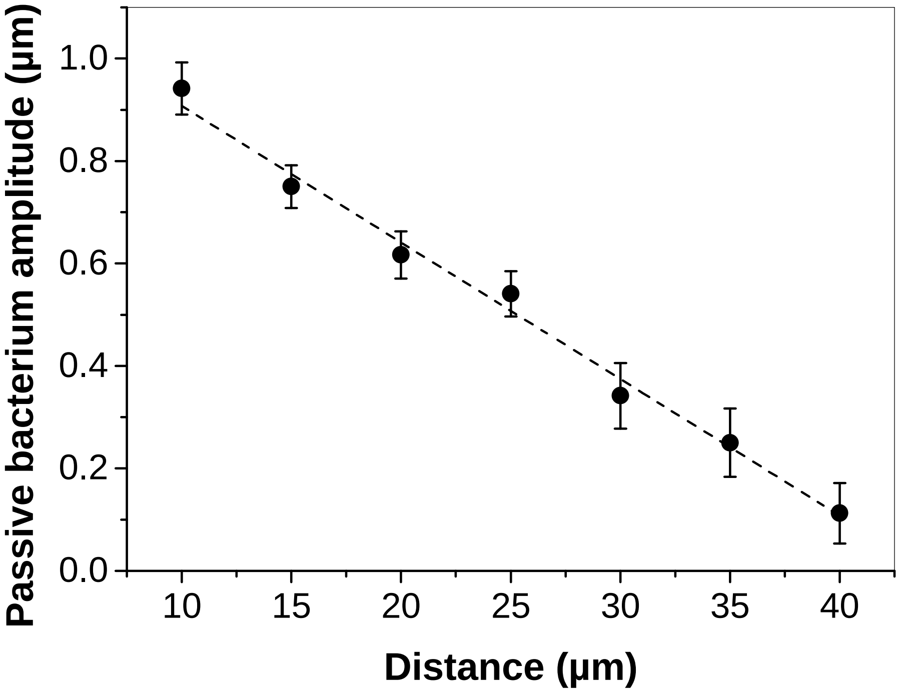


**Supplementary Figure 3. Distance dependence of the bacterial coupling strength.** *B. subtilis* wt was grown for 2.5 h in SYM growth medium at 28 °C, 200 rpm. Amplitude of the active bacterium was 3 µm, $\nu = 0.5$ Hz. The average values and standard errors are given ($n = 8$). Line represents linear fit to the data ($r^2 = 0.98637$).

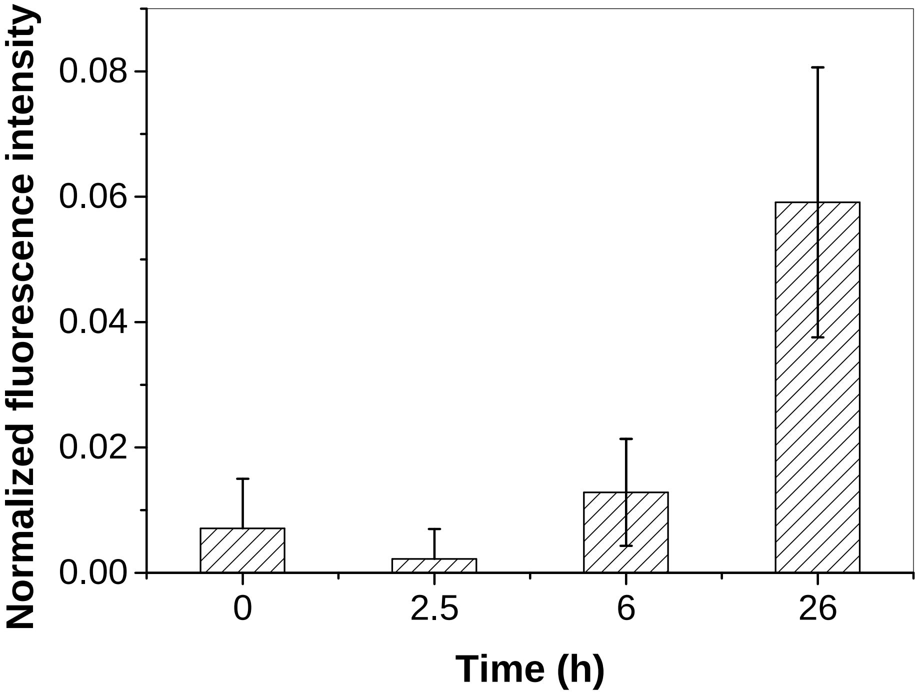


**Supplementary Figure 4. The expression of $P_{epsA}$-*gfp* gene construct.** The average normalized single cell fluorescence intensity of *B. subtilis* YC164 cells expressing $P_{epsA}$-*gfp* reporter corrected for the background. Average values and standard errors are given (*n* = 4).

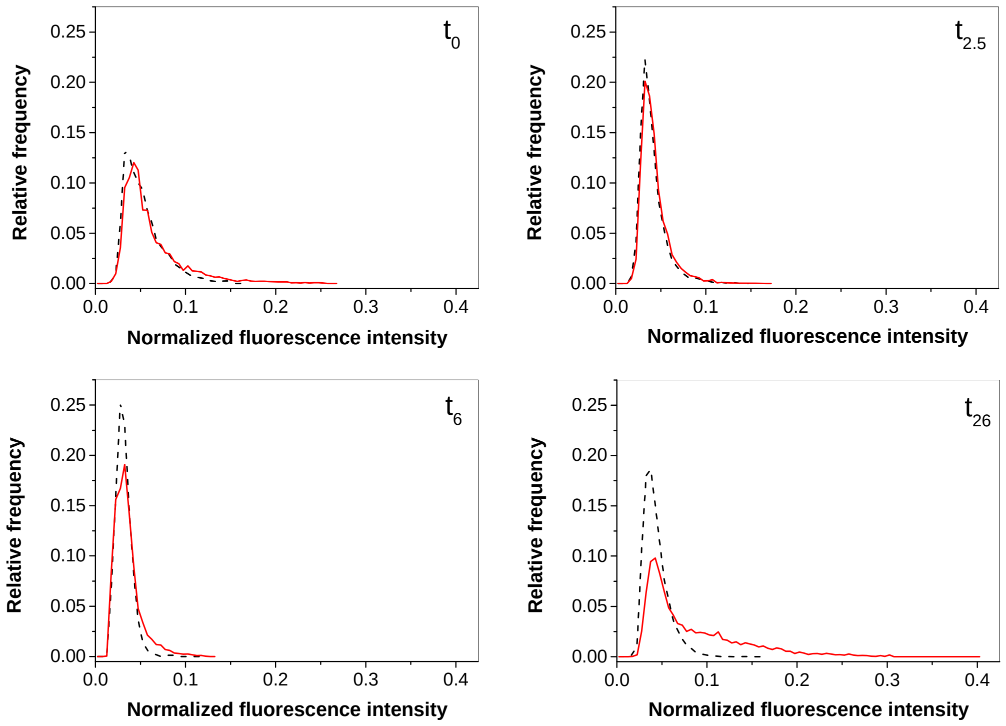


**Supplementary Figure 5. Single cell fluorescence intensity distribution during *B. subtilis* growth.** Relative normalized single cell fluorescence distribution of *B. subtilis* wt autofluorescence (- - -) and *B. subtilis* YC164 expressing $P_{epsA}$-*gfp* gene construct (——) at 0, 2.5, 6 and 26 h of incubation. Average values are given (*n* = 4).

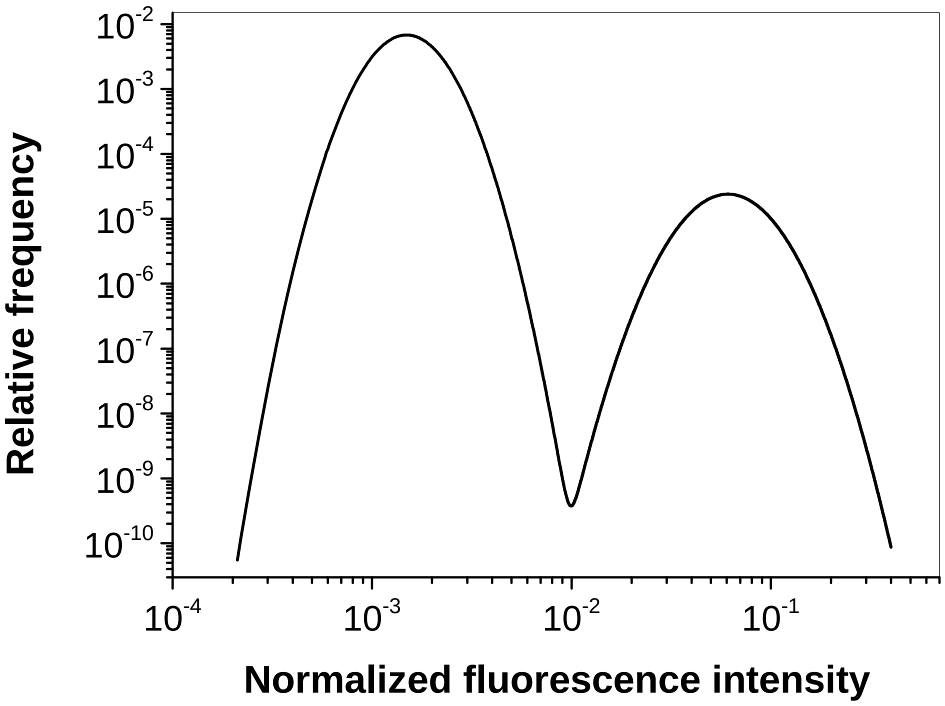


**Supplementary Figure 6. Biphasic expression of P$_{epsA}$-*gfp* gene construct.** The distribution of *B. subtilis* YC164 single cell normalized fluorescence intensity. Two subpopulations with P$_{epsA}$-*gfp* gene construct of different fluorescence intensity (low and high) after 26 h of growth are depicted. Average values are given (*n* = 4).

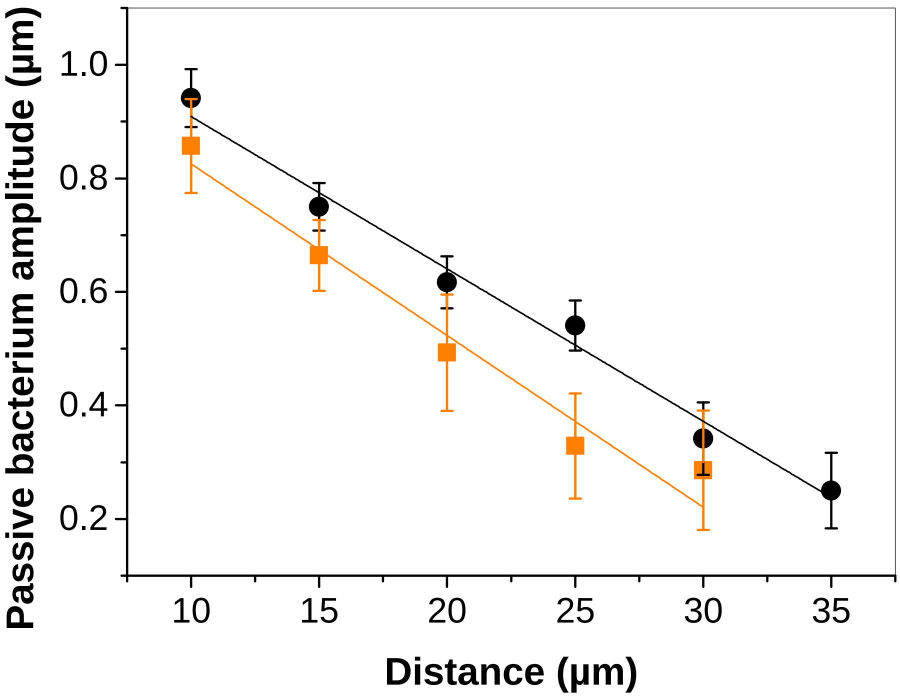


**Supplementary Figure 7. The effect of *eps*(*A-O*)$^-$ *tasA*$^-$ double mutant on the mechanical coupling.** The strength of the mechanical coupling of the *B. subtilis* wt (●) and *eps*(*A-O*)$^-$ *tasA*$^-$ double mutant (■) with increasing distance. Bacterial cultures were grown for 2.5 h in SYM medium at 28 °C, 200 rpm. Lines represent linear fit to the data. The average values and standard errors are given (*n* = 7 or more).

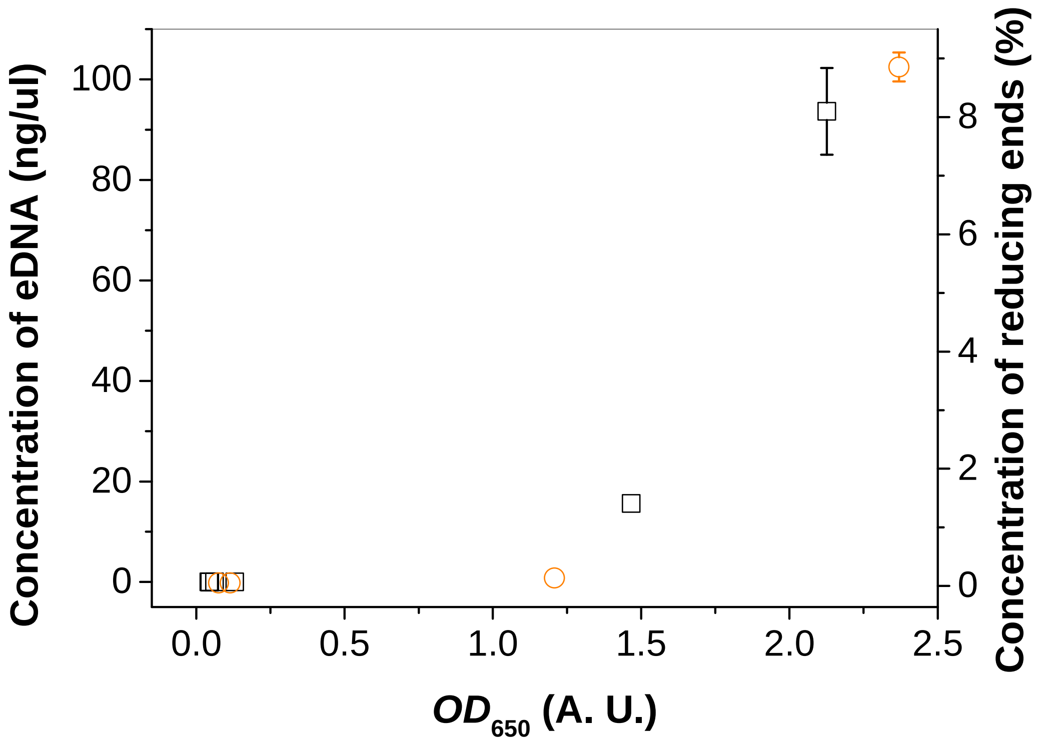


**Supplementary Figure 8. The early extracellular network contains low amount of eDNA and reducing sugars.** Concentrations of eDNA (□) and reducing sugars (○) during the growth of *B. subtilis* wt. The average values and standard deviations are given ($n$ = 3).

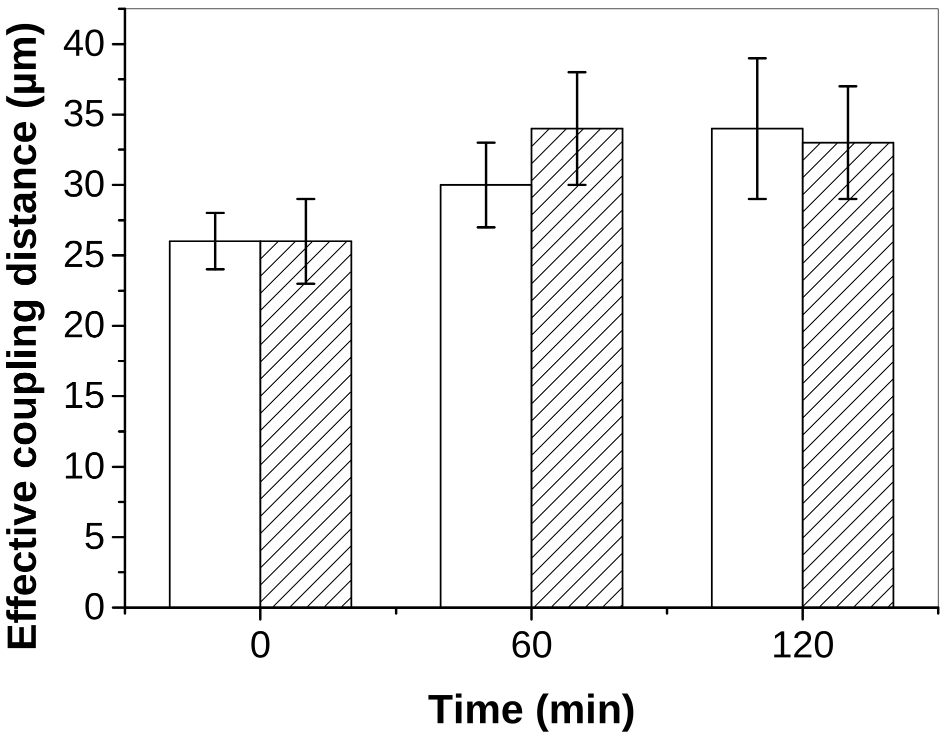


**Supplementary Figure 9. The effect of sodium azide on the mechanical coupling.** Sodium azide was added to the *B. subtilis* grown in SYM at different time of incubation (0, 60 and 120 min). Samples were incubated in growth medium with 7.7 mM final concentration of sodium azide and the mechanical coupling was measured prior to the addition (white bars) and at the end of incubation in sodium azide (hatched bar). The average values and standard deviations are given ($n$ = 4).

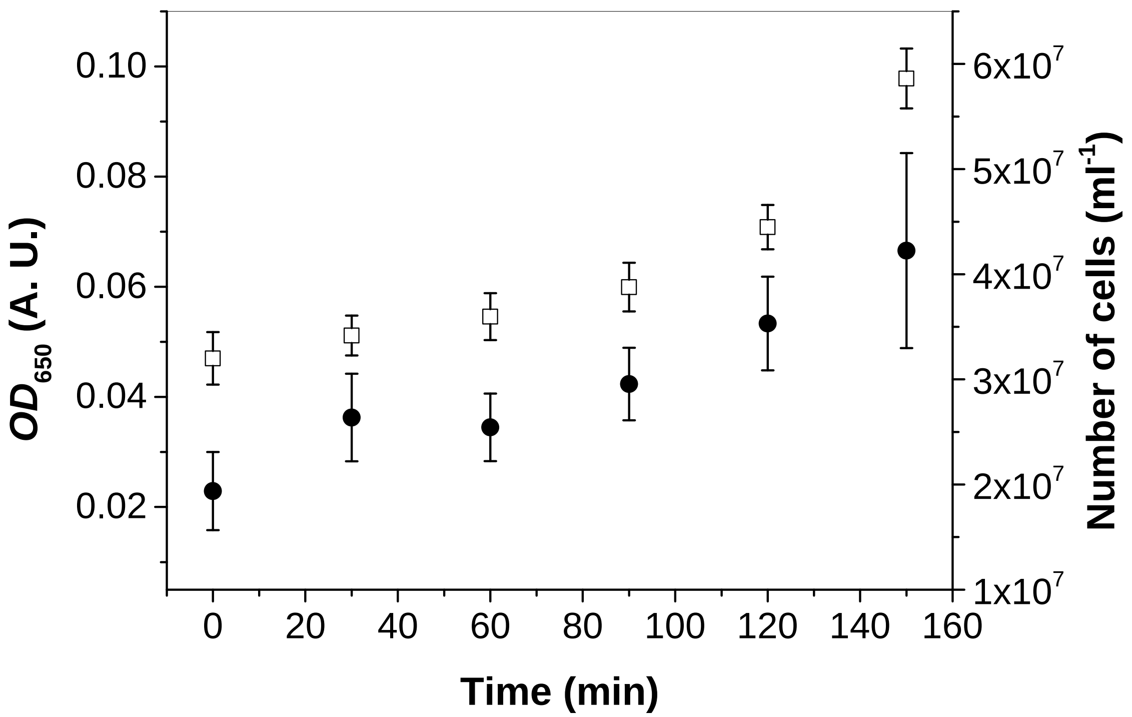


**Supplementary Figure 10. Low density bacterial growth.** Growth of *B. subtilis* wt in SYM growth medium in a diluted cell suspension, during 150 min of incubation. (□) represents optical density ($n$ = 10 or more), (●) depicts number of cells as determined by microscopy ($n$ = 4). The average values and standard errors are given.

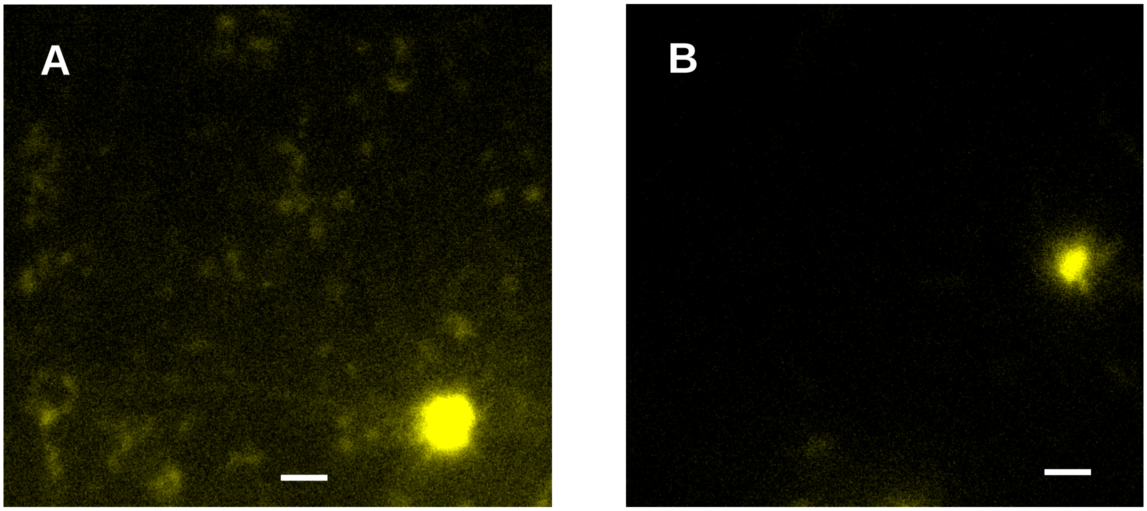


**Supplementary Figure 11. The presence of eDNA in bacterial inoculum.** Fluorescence microscopy of overnight culture *B. subtilis* culture stained with nanosensitive TOTO-1 (**A**), and overnight culture washed in saline solution and re-suspended in SYM growth medium (**B**). Bright object is a lysed bacterium. Intact bacteria impermeable to TOTO-1 representing the majority of cells are not visible on the micrographs. Scale bars represent 3 μm.

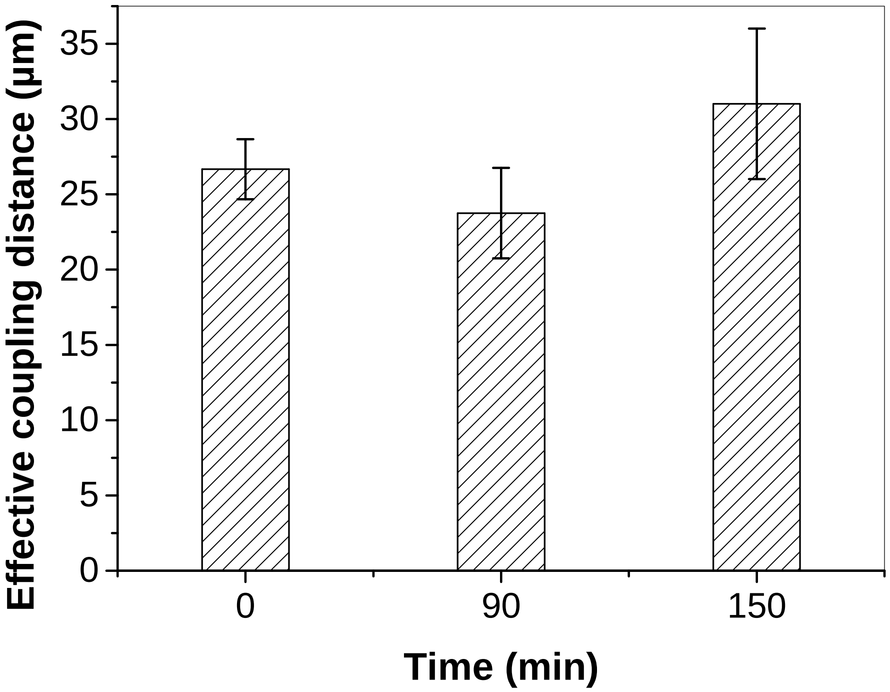


**Supplementary Figure 12. The mechanical coupling of stationary** *B. subtilis* **cells in PBS.** Mechanical coupling of the stationary *B. subtilis* cells washed, re-suspended, and incubated in the PBS medium for 2.5h. The average values and standard deviations are given ($n$ = 3).

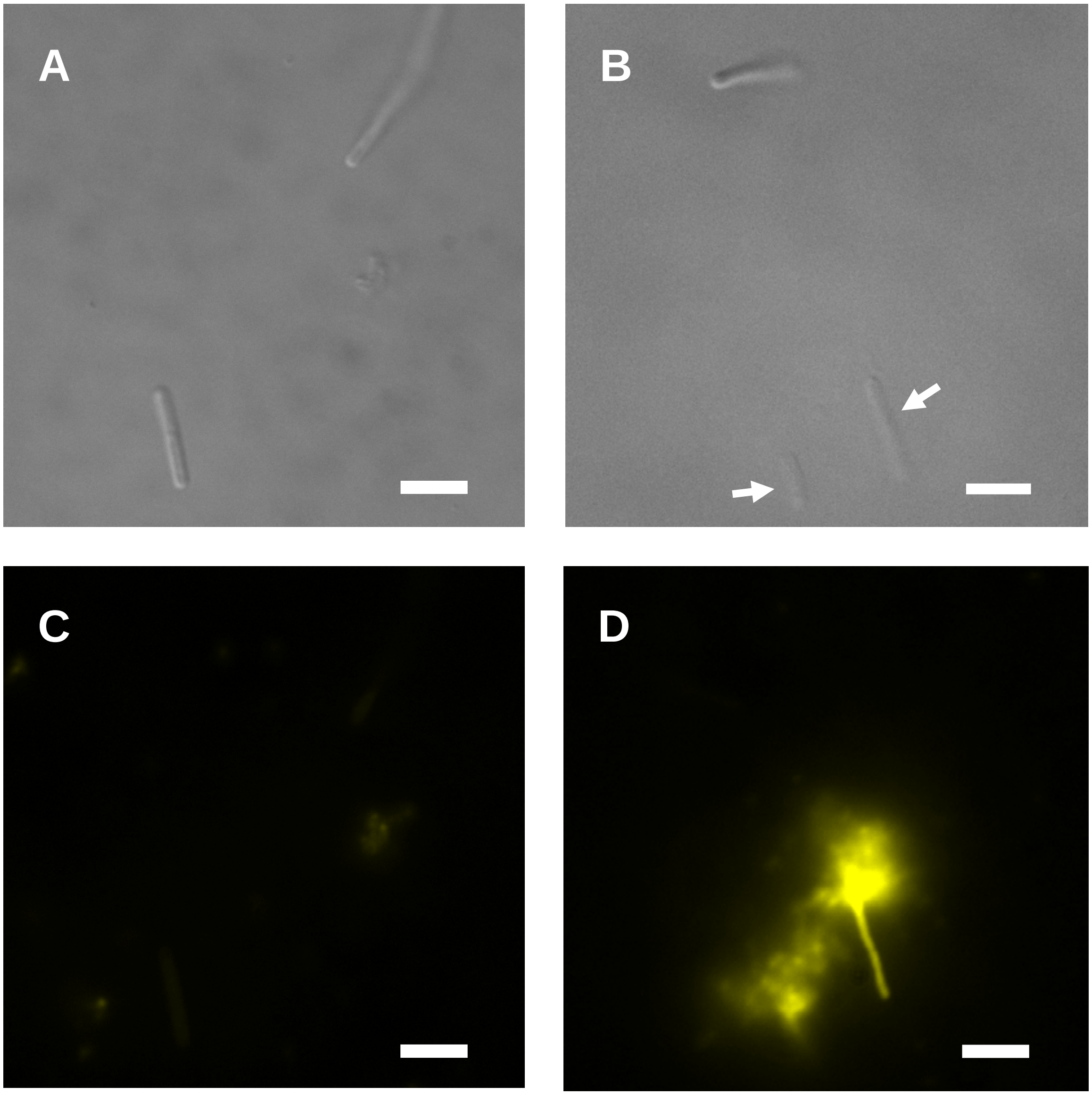


**Supplementary Figure 13. The release of nucleic material during cell lysis of exponentially grown cells.** Top row: DIC microscopy of *B. subtilis* wt samples after 2 min (**A**) and after 60 min (**B**). Bottom row TOTO-1 fluorescently stained cells after 2 min (**C**) and after 60 min (**D**). The same view field is shown at a given time for DIC and fluorescence images. Arrows point to morphologically deflated cells that have intense fluorescence with small fluorescence particles diffusing away from the lysed cells. Scale bars represent 5 µm.

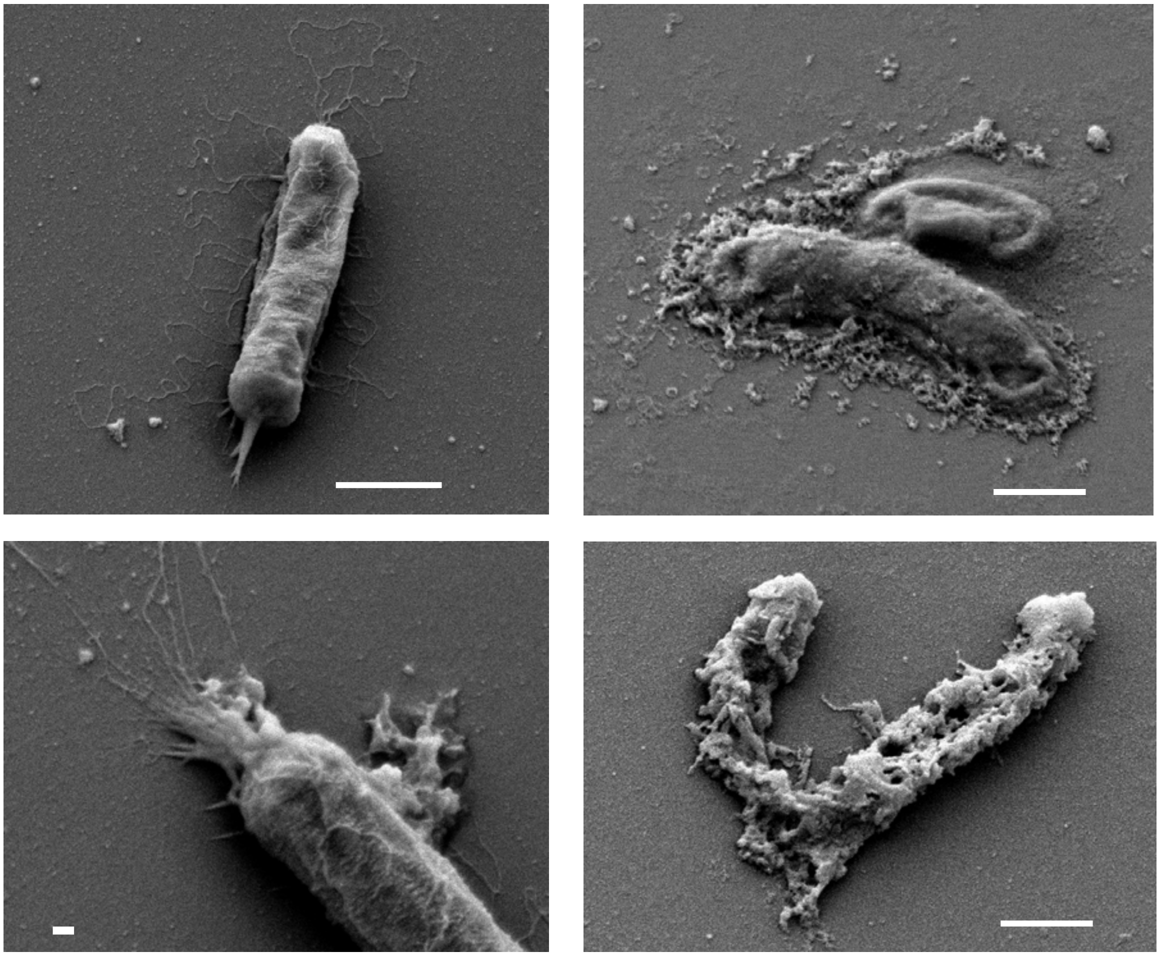

**Supplementary Figure 14. A progressive morphological decay during cell lysis.** SEM micrographs during cell lysis of the exponentially grown *B. subtilis* cells after 60 min in SYM medium at rest (without shaking). Large scale bars represent 1μm, small scale bar represents 0.1 μm.

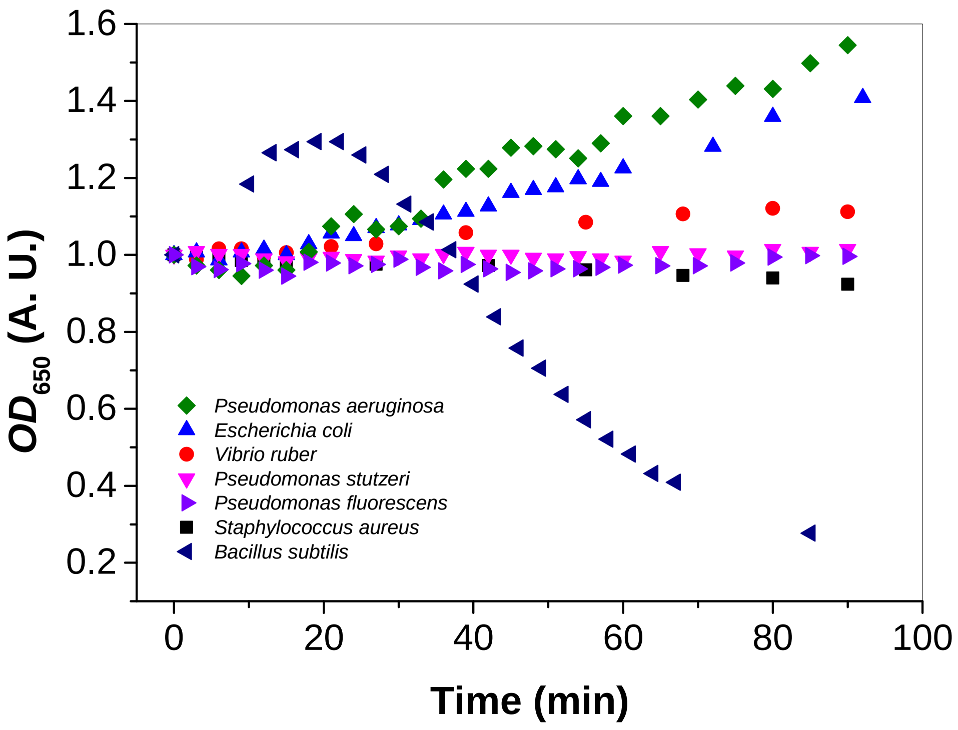


**Supplementary Figure 15. Cell lysis of exponentially grown bacteria.** Cells were grown to $OD_{650}$ ~ 0.5 when put to rest. Due to visible aggregate formation in *P. aeruginosa* lysis experiment was performed in the early exponential phase at $OD_{650} = 0.2$. *P. aeruginosa* (◆), *E. coli* (▲), *V. ruber* (●), *P stutzeri* (▼), *P. fluorescens* (▶), *S. aureus* (■), and *B. subtilis* (◀). Optical densities were normalized to the initial optical density.

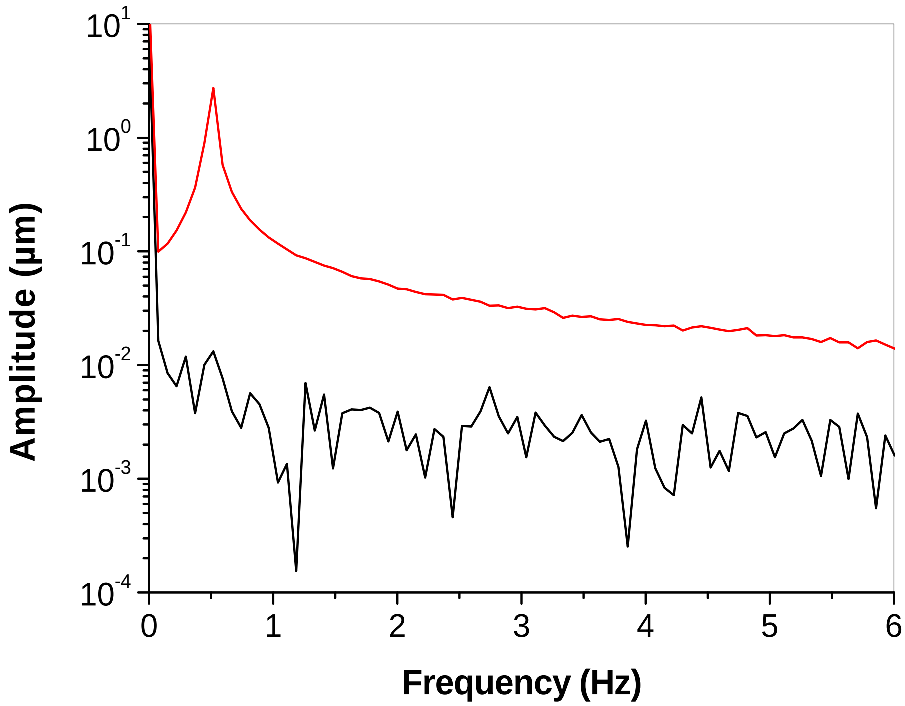


**Supplementary Figure 16. The correlated motion of optically trapped bacterial pairs.** Fast Fourier transformed data of the correlated motion of optically trapped active (red line) and passive (black line) *B. subtilis* wt bacterial pairs in PBS suspension at a distance of 10 µm. There was no noticeable coupling of the passive bacterium motion at the frequency of active bacterium at $\nu = 0.5$ Hz suggesting the absence of hydrodynamic effect in simple solutions.

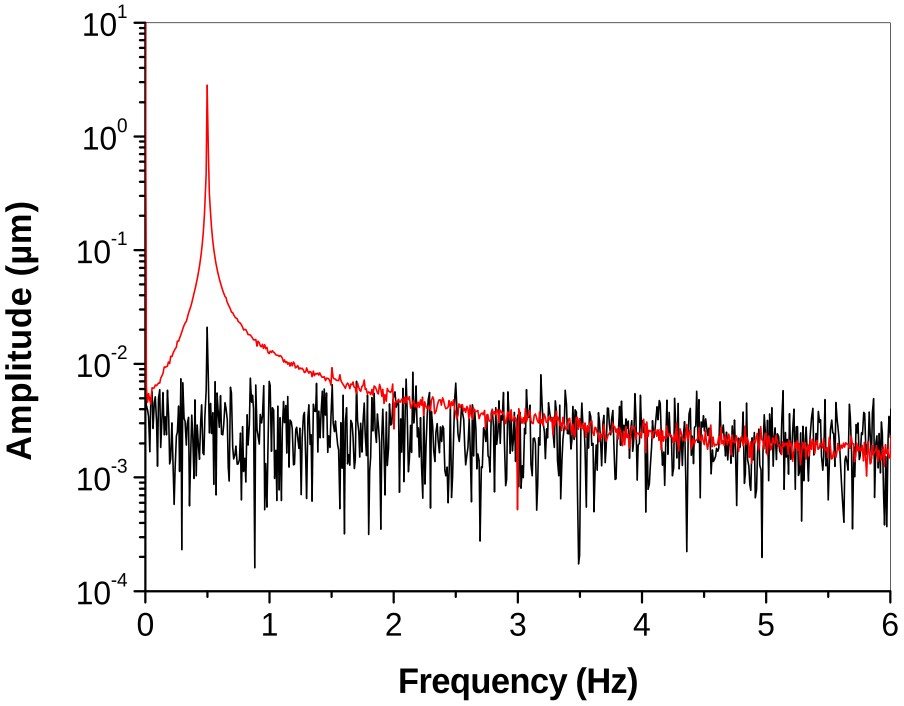


**Supplementary Figure 17. The correlated motion of optically trapped silica beads.** Fast Fourier Transform data of the correlated motion of the active (red line) and passive (black line) silica beads ($a$ = 2.32 μm) in $dH_2O$ at distance of 10 μm. The results suggest that detectable range of hydrodynamic effect of smooth surface particles is less than 10 μm.

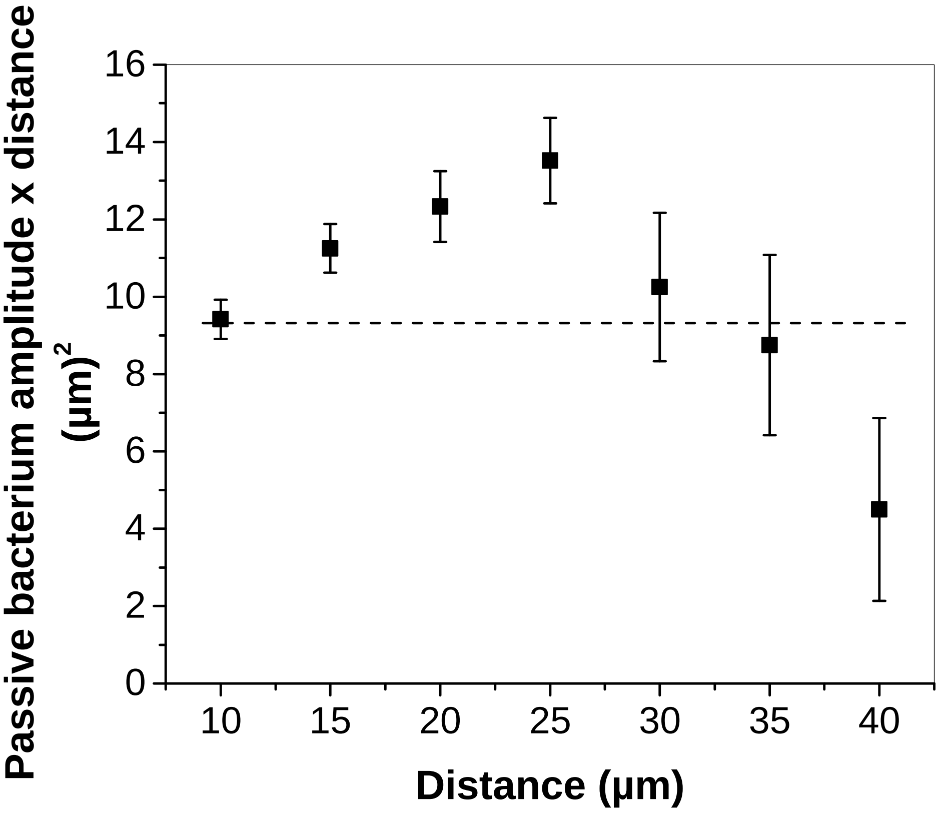


**Supplementary Figure 18. The long-range mechanical coupling and the hydrodynamic effect.** A plot of the passive bacterium amplitude response *A* multiplied by the inter-bacterial distance *r* as a function of the inter-bacterial distance *r*. The dashed line represents the case of hydrodynamical forcing with no typical coupling distance. The data are extracted from Supplementary Fig. 3. The average values and standard deviations are given (*n* = 8).

**Supplementary Table 1. Development of the mechanical coupling in dilute *B. subtilis* wt suspensions.**

| | **Passive bacterium amplitude (μm)** | | | | | |
|---|---|---|---|---|---|---|
| **Distance (μm)** | **after inoculation** | **after 30 min** | **after 60 min** | **after 90 min** | **after 120 min** | **after 150 min** |
| **10** | 0.8 ± 0.1 | 1.0 ± 0.2 | 0.8 ± 0.1 | 0.9 ± 0.1 | 1.0 ± 0.1 | 0.9 ± 0.1 |
| **15** | 0.6 ± 0.1 | 0.7 ± 0.1 | 0.7 ± 0.1 | 0.7 ± 0.1 | 0.7 ± 0.1 | 0.8 ± 0.1 |
| **20** | 0.4 ± 0.1 | 0.4 ± 0.2 | 0.5 ± 0.3 | 0.5 ± 0.2 | 0.5 ± 0.1 | 0.6 ± 0.1 |
| **25** | | | 0.4 ± 0.2 | 0.2 ± 0.2 | 0.4 ± 0.1 | 0.5 ± 0.1 |
| **30** | | | | | 0.2 ± 0.1 | 0.3 ± 0.1 |
| **35** | | | | | | 0.3 ± 0.1 |

The strength of *B. subtilis* wt mechanical coupling, determined as the amplitude of the passively trapped bacterium, measured at different distances from the actively trapped bacterium and at different growth times. The average values and standard errors are given ($n$ = 4 or more).
